# *In Vivo* Anticancer Activity of* Basella alba* Leaf and Seed Extracts against Ehrlich's Ascites Carcinoma (EAC) Cell Line

**DOI:** 10.1155/2018/1537896

**Published:** 2018-11-19

**Authors:** Md. Shihabul Islam, Md. Sifat Rahi, Chowdhury Arif Jahangir, Md Habibur Rahman, Israt Jerin, Ruhul Amin, Kazi Md. Faisal Hoque, Md Abu Reza

**Affiliations:** ^1^Molecular Biology and Protein Science Laboratory, Department of Genetic Engineering and Biotechnology, 3rd Science Building, Level No. 4, University of Rajshahi, Rajshahi 6205, Bangladesh; ^2^Bangladesh Council of Scientific and Industrial Research, Laboratory, Rajshahi 6206, Bangladesh

## Abstract

Cancer is a class of diseases characterized by uncontrolled cell growth. The current treatment options of cancer are radiotherapy, chemotherapy, hormone therapy, and surgery, where all of them have unpleasant side effects. Due to their adverse side effects, it is challenging to develop new drug for cancer treatment. Hence, the scientists are trying to seek for noble compounds from natural sources to treat cancer. Therefore, in the present investigation, a widely consumable vegetable* Basella alba* was subjected to evaluate its antiproliferative effect along with molecular signaling of apoptosis in Ehrlich ascites carcinoma (EAC) cell line. Cell growth inhibition was determined by haemocytometer whereas apoptosis of cancer cells were studied by florescence microscope using Hoechst-33342 stain and result was supported by DNA fragmentation and certain cancer related genes expression through PCR analysis.* B. alba* leaf and seed extract exhibit a considerable scavenging activity in comparison to a standard antioxidant BHT. Moreover, the leaf and seed extracts were able to agglutinate 2% RBC of goat blood at minimum 12.5*μ*g/ml and 50.0*μ*g/ml concentration, respectively. A significant cytotoxic activity was also found in both leaf and seed extract. In haemocytometic observation, the leaf and seed extracts exhibit about 62.54±2.41% and 53.96±2.34% cell growth inhibition, respectively, whereas standard anticancer drug Bleomycin showed 79.43±1.92% growth inhibition. Morphological alteration under fluorescence microscope showed nuclear condensation and fragmentation which is the sign of apoptosis. Apoptosis induction was also confirmed by DNA laddering in leaf and seed treated EAC cells. Upregulation of the tumor suppressor gene P^53^ and downregulation of antiapoptotic gene Bcl-2 enumerate apoptosis induction. Therefore, current study manifested that leaf and seed extracts of* B. alba *have antiproliferative activity against EAC cell line and can be a potent source of anticancer agents to treat cancer.

## 1. Introduction

Cancer is considering the burning health issue and is one of the most life threatening diseases in both developed and developing countries right now [1]. Blood cancer, lung cancer, breast cancer, prostate cancer, cervix cancer, bone cancer, ascites cancer are thought to be most occurring cancer around the globe and all these cancers can cause death [2]. It is a group of diseases caused by loss of cell cycle control leading to abnormal and uncontrolled cell growth [3]. Cancer development is associated with the alteration of oncogenes, tumor suppressor genes, and DNA repair genes [4]. Both external factors such as tobacco, chemicals, radiation, and infectious organisms and internal factors such as inherited mutations, hormones immune conditions are considered to be responsible or the risk factors for causation of cancer [5].

Cancer imposes a serious burden on the public health and its treatment and curing processes are still scientifically challenging [6]. The conventional approaches of cancer treatments are chemotherapy, radiotherapy, hormone therapy, and surgery. But each of these conventional treatment modules has severe side effects [7]. The increased death incidence and the adverse effects of anticancer drugs are the main reasons that motivated the researchers to look for new and more effective drugs with lesser side effects [8]. Due to these limitations, scientists are in constant search of natural compounds which might be capable of healing cancer [9].

Many natural compounds such as terpenoids, phenolic acids, lignans, tannins, flavonoids, quinones, coumarins, and alkaloids were discovered from plant sources that contain significant antioxidant activities and play an important role in cancer treatment [10]. Several studies manifested that the antioxidant compounds show anti-inflammatory, antitumor, antimutagenic, and anticarcinogenic activities [11]. Natural compounds with antioxidant activity can directly inhibit cell proliferation and stimulate the immune system [12].

In recent year, the drug industries largely depend on natural compounds as a source of medicine. The statistics showed that over 60% of the recently used anticancer drugs are related to herbal origin [13]. Herbal products are worldwide accepted as a source of complementary and alternative medicine in [14] various diseases especially in cancer [15]. They provide us relatively safe, effective, and economical therapeutic options, particularly in case of cancer where treatment is long term and cost is excruciatingly high [16].

Due to the favorable climatic condition, Indian subcontinent is the home of wide range of plant species with medicinal properties. Ayurveda is one of the most ancient medicine systems which are developed in India with a fundamental principle [17]. This treatment system comes out based on the plant's materials and is running smoothly from the very ancient time till today. The history of Ayurveda reported that it gains an excellent achievement in the world medicine. Despite the modern medicine achieves a remarkable consequences through physical, chemical, and natural sciences, the Ayurvedic medicine shows a fruitful contribution to remission of human sufferings [18]. So, the current focus is to identify many bioactive compounds with anticancer potentialities for combating cancer.

*B. alba* is a fast grown leafy vegetable plant with high medicinal values which is cultivated worldwide [19]. It contains fiber, ash, calcium, vitamins, thiamine, riboflavin, niacin, etc. and is traditionally used as an antidote, aperients, astringent, demulcent, diuretic, febrifuge, laxative, and rubefacient [20, 21]. It also works well in the treatment against inflammation, atherosclerosis, stroke, heart disease, diabetes mellitus, multiple sclerosis, Parkinson's disease, Alzheimer's disease, etc. [22]. Moreover, leaves of the plant exhibited its potentiality of recovering male infertility [23]. Therefore, present study was designed to investigate the anticancer potential of leaf and seed extracts of* B. alba* thorugh* in vivo *mouse model.

## 2. Materials and Methods

### 2.1. Collection and Preparation of Leaf and Seed Extracts

Malabar Spinach/Bangladeshi Pui shak was one of the kinds of* B. alba* used in this experiment. The plant materials (*B. alba *leaf and seed) were collected from the botanical garden of Botany Department, University of Rajshahi, and washed thoroughly with distilled water. Plant specimens were authenticated by Dr. A. H. M Mahbubur Rahman, Professor, Department of Botany, University of Rajshahi, Bangladesh. About 500g of fresh leaf and 500g of seed were dried at 40°C for 10 days and 25 days respectively. Upon drying, the plant materials were powdered with a power grinder (Jaipan, India). About 25g of both powdered leaf and seed were dissolved in 250 ml of distilled water in separate conical flask and overnight shaking was applied using a rotator (Digital rotator, Taiwan). Subsequently, the samples were sonicated at medium frequency for 15 min in an ultrasonic bath (Soniprep 150, UK) and filtered with Whatman No.1 filter paper using vacuum pump filtration system. The filtered materials were lyophilized using freeze dryer and stored at 4°C until further used.

### 2.2. Experimental Animal

Healthy Swiss Albino mice weighing 25 ± 2.0 g were collected from the animal house of Pharmacy Department, University of Jahangirnagar, Dhaka, Bangladesh. The mice were clustered into three groups (control, leaf treated, and seed treated) containing 6 mice in each group. They were housed in plastic cage and maintained with standard condition (25 ± 2°C) with 12 ± 1-hour dark/light cycle. Mice were fed with pellet feed containing proper nutrients.

### 2.3. Collection and Maintenance of Cell Line

The initial inoculums of Ehrlich ascites carcinoma (EAC) cells were kindly provided by Protein and Enzyme Laboratory, Department of Biochemistry and Molecular Biology, University of Rajshahi, Bangladesh. The EAC cells culture and aspiration were maintained following the procedure of Alam et al. [24] with minor modification. In brief, the EAC cells were thereafter propagated intraperitoneal (i.p.) in our laboratory biweekly by injecting cells, freshly drawn from a donor Swiss Albino mouse bearing 6-7-day-old ascites tumor. The freshly drawn fluid was diluted with normal saline (1%NaCl solution). The aspirated cells were kept in a cell culture petridish for 1 hour at 37.5°C in an air incubator. All macrophages, as distinct from tumor cells, became firmly fixed to the bottom of the culture vessels [25]. Then, the petridish was briefly vortexed and the fluid was collected for EAC cells which were used in subsequent experiments. The tumor cells number was adjusted to approximately 2×10^6^ cells/ml by counting the cell number with the help of a haemocytometer. The viability of tumor cells was observed by trypan blue dye (0.4%) exclusion assay. Strict aseptic condition was maintained throughout the transplantation process.

### 2.4. Ethical Clearance

This research work was approved by the Institutional Animal, Medical Ethics, Bio-Safety and Bio-Security Committee (IAMEBBC) for Experimentations on Animal, Human, Microbes, and Living Natural Sources, memo no. 31/320-IAMEBBC/IBSc, Institute of Biological Sciences, University of Rajshahi, Bangladesh.

### 2.5. Chemicals and Reagents

Hemagglutination buffer, Saline water, Sodium citrate (anticoagulant), 1, 1-diphenyl-2- picrylhydrazyl (DPPH), Methanol, Ethanol, Butylated hydroxyl toluene (BHT), Phosphate buffer saline (PBS), Hoechst-33342 dye, TIANamp Genomic DNA kit (Tiangen, Beijing, China), RNAsimple Total RNA kit (Tiangen, Beijing, China), Agarose, Ethidium bromide, TBE buffer, Trypan blue, Benedicts reagents, Biuret reagents, Wagner's reagents, HCl, FeCl_3_, H_2_SO_4_. All the used chemicals and reagents were of laboratory and analytical grade.

### 2.6. Phytochemical Screening

Standard procedures were used to screen the presence of different phytochemicals from leaf and seed of* B. alba. *The crude extracts of leaf and seed of* B. alba *were tested for the presence of carbohydrates (Benedict's test) [26], proteins (Biuret test) [27], fats (Filter paper test) [28], glycosides (Salkowski's test) [29], alkaloids (Wagner's test) [30], tannins (Ferric chloride test) [31], phlobatannins (HCl test) [32], saponins (Frothing test) [33], flavonoids (HCl test) [34], steroids (Salkowski's test) [32], phenolic compounds (ferric chloride test) [29], and phytosterols (sulfuric acid test) [35]. The qualitative results are expressed as (+) for the presence and (-) for the absence of phytochemicals.

### 2.7. Antioxidant Activity Assay

The DPPH scavenging activity of leaf and seed extracts was determined using the method described previously by Brand-Williams et al. in 1995 with slight modification in microtiter plate [36]. According to this, 1 ml of methanolic solution of leaf and seed extracts were taken in ten (10) test tubes containing different concentrations such as 20, 40, 60, 80, 100, 120, 140, 160, 180, and 200*μ*g/ml from stock solution (1 mg/ml). Subsequently, 1.5 ml DPPH (1mg/25 ml) was added to these test tubes and the mixtures were allowed to keep at room temperature in the dark place for 30 minutes. Finally, absorbance was measured at 517 nm using spectrophotometer (GENESYS 10S UV-Vis, Thermo Scientific, USA). BHT was used as standard and methanol as negative control. The percentage of DPPH scavenging capacity was calculated according to the formula below:

(1) $$\begin{array}{c} DPPH\;\;scavenging\left(\;\%\;\right)=\frac{C-E}{C}\times 100\% \end{array}$$

Where, C is absorbance of the control and E is absorbance of the leaf and seed extracts.

The 50% inhibition concentrations of the extracts, IC_50_, values were determined by plotting the graph of percentage DPPH scavenging activity against the different concentrations of the extracts.

### 2.8. Hemagglutination Assay

The hemagglutination assay of crude leaf and seed extracts of* B. alba* were performed in 96-well microtiter U-bottomed plates following the procedure described by Hasan I. et al., 2014 [37]. According to this process, goat blood was collected in test tubes containing sodium citrate (anticoagulant reagent) and washed for 2-3 times with PBS (phosphate buffer saline) to prepare 2% (w/v) red blood cells. This 2% RBC was prepared followed by a standard process described by Rashel Kabir et al. (2012) [38]. In brief the goat blood was collected from slaughtered house in sodium citrate and centrifuged at 3000 rpm for 5 minutes at 4°C to separate the blood cells from plasma. Then, the cell was washed for three times with PBS (phosphate buffer saline). After that, 20 mg of RBC pellet was taken and dissolved in 1mL of hemagglutination buffer. Subsequently, 50*µ*l of hemagglutination buffer was taken in each well of microtiter plate. Instantly, 50*µ*l of leaf and seed extracts were added to the first well and serially diluted. Finally, 50*µ*l of 2% red blood cells were added to each well of the titer plate after 30 minutes of shaking; the agglutinating activity was measured.

### 2.9. Cytotoxicity Assay

It was carried out by using brine shrimp (*Artemia salina*) nauplii hatched after 48 hours in saline water (1% NaCl). For this experiment, first ten (10) different concentrations (25, 50, 75, 100, 125, 150, 175, 200, 225, and 250 *µ*g/ml) from prepared stock solution (1mg/ml) were taken in different ten (10) test tubes and made the total volume up to 10 ml by adding 1% saline. Afterwards, 20 pieces of hatched shrimps were taken in each test tube and kept at room temperature for observation. The live and dead shrimps were counted after 24 hours and the LC_50_ value was calculated with the help of regression line using the obtained data in Microsoft Excel 2007.

### 2.10. Evaluation of Weight Loss

To evaluate the weight loss, after inoculation of carcinoma cells, weight of each mouse was measured by electric balance (KAMRY, electronic kitchen scale, China) for six consecutive days during treatment period. Finally the rate of weight loss was calculated by using the average value for both control and treated mice using the following equation:

(2) The  rate  of  weight  loss=1st  day  weight−Last  day  weightTotal  day  of  treatment  gm/day

### 2.11. Cell Growth Inhibition

The evaluation of* in vivo* cancer cell growth inhibition was conducted by using a common and simple process described by Sur P. et al., 1994 [39]. To determine the cell growth inhibition, four groups (control, Bleomycin, leaf, and seed) of Swiss Albino mice (6 in each group) were used where 1.72 × 10^6^ EAC cells per ml were inoculated in every mouse of each group. After 24 hours, 5.00 mg/kg/body weight leaf and seed extract were administrated for therapeutic evaluation and continued for six days. Mice in each group were sacrificed on day seven when the total intraperitoneal EAC cells were isolated and diluted in normal saline (1% NaCl). Viable cells were first counted by haemocytometer using trypan blue stain through the following equation:

(3) Cells/ml=The  average  count  per  square×Dilution  factorDepths  of  fluid  under  cover  slip×Area  counted

Percentage of cell growth inhibition was calculated by comparing the total number of viable cells in the treated groups with control group using the following equation.

(4) Percentage  of  cell  growth  inhibition%=1−TwCw×100%

Where, Tw is mean of number of EAC cells of the treated group mice and Cw is mean of number of EAC cells of the control group mice.

### 2.12. Study on Apoptosis

The apoptotic cells were identified through the morphological alteration or hallmark features during microscopic observation either by light or by fluorescence microscope [40]. Study of cellular apoptosis was carried out by observation of morphological changes of EAC cells from both control and treated mice using a fluorescence microscope (Olympus iX71, Korea). In brief, EAC cells were collected from both control and treated mice after 6 days of treatment and the cells were washed for 2-3 times with phosphate buffer saline (PBS). Subsequently, the cells were stained with 1mg/1.6ml of Hoechst-33342 solution (20mM) dye at 37°C for 20 min. Finally, the cells were washed with phosphate buffer saline (PBS) and resuspended in PBS for observation of morphological changes under fluorescence microscope.

### 2.13. DNA Laddering

The DNA laddering is an important feature of the apoptotic cells and this study was carried out by following the procedure described by Andrew Wyllie AH et al., 1980 [41], to observe the cellular apoptosis as well as nucleotide cleavage. EAC cells were collected from the both control and treated mice and the total genomic DNA was extracted using TIANamp Genomic DNA kit (Tiangen, Beijing, China) according to manufacturer's protocol. DNA was run on 1% agarose gel containing 0.1% ethidium bromide. After 50-60 minutes of running at 100 volt, the gel was visualized under UV light using gel documentation system (Proteinsimple, Alphaimager Mini, USA).

### 2.14. Gene Expression Analysis

Total RNA from both treated and nontreated (control) EAC cells was isolated using RNAsimple Total RNA kit (Tiangen, Beijing, China) following manufacturer's guidelines. The quality and quantity of the RNA was measured by nanodrop (Thermo scientific). The cDNA was prepared through reverse transcription PCR (polymerase chain reaction) using 3*µ*g total RNA, 2 *µ*l of 10mM oligo dT, 1 *µ*l of 10mM dNTPs, and 1 *µ*l of TIANscript MMLV reverse transcriptase (Beijing, China).

Expression of apoptosis related gene such as P^53^ and Bcl-2 was studied by PCR where GAPDH, a housekeeping gene, was used as control. The sequences of primer used in the experiment are shown in Table 1. Each 10 *µ*l of PCR reaction mixture contained 0.40 *µ*l of 10 mM dNTPs, 0.50 *µ*l templates, 0.40 *µ*l each of 5mM forward and reverse primer, 0.10 *µ*l of polymerase, and 1*µ*l of 10X DNA polymerase buffer and topped up with deionized water.

Reaction conditions were initial PCR activation step of 3 min at 95°C, followed by 35 cycles of 95°C for 45 sec, 52°C for 45 sec, and 72°C for 1.00 min and a final extension of 72°C for10 min. PCR reactions were analyzed on 1 % agarose gel using Tiangen 1kb plus DNA ladder (Tiangen, Beijing, China) as DNA marker.

### 2.15. Statistical Analysis

All the statistical analyses were carried out in triplicate. Data are expressed as mean ± SD. The test of significance was performed by SPSS-16 using one-way ANOVA followed by a Dunnett post hoc test compared with control. The significant levels were set up at 5%, 1%, and 0.1% level where P∗<0.05, P∗∗<0.01, and P∗∗∗<0.001, respectively. Microsoft Excel 2007 was used for the statistical and graphical presentation of data.

## 3. Results

### 3.1. Phytochemical Screening

The qualitative phytochemical constituents of leaf and seed extract of* B. alba* are shown in Table 2. Analysis revealed the presence of alkaloids, saponins, tannins, flavonoids, glycosides, steroids, phenolic compounds, and phytosterols whereas phlobatannin was not detected in either leaf or seed extract. Proteins, carbohydrates, and fats were found in seed in higher quantity than that of leaf. On the contrary, glycosides, tannin, and phenolic compounds were present in higher amount in leaf more compared to seed. The sign “+” and “–” represent the presence and absence of the particular compounds.

### 3.2. Antioxidant Activity

**Figure 1:**
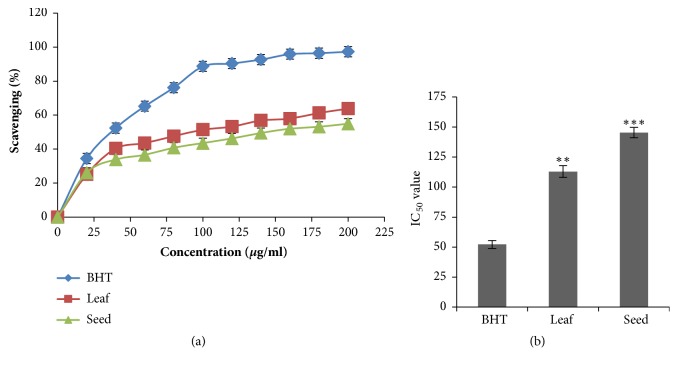
The antioxidant activity assay of leaf and seed extracts of* B. alba* along with standard BHT. (a) Scavenging activity of leaf and seed extracts along with standard BHT. (b) Comparison of IC_50_ values of leaf and seed extracts with standard BHT. Each value is expressed as mean ± SD (n=3) and significance was set at P<0.05 (*∗*), P <0.01 (*∗∗*), and P <0.001 (∗∗∗) compared to standard.

The antioxidant activity of* B. alba* was evaluated using DPPH free radical scavenging assay. DPPH free radical scavenging activity of leaf and seed of* B. alba* along with standard BHT is shown in Figure 1(a). The IC_50_ value of leaf and seed of* B. alba* and standard BHT were calculated as 112.96±4.87*µ*g/ml, 145.34±4.38*µ*g/ml, and 52.27±3.31*µ*g/ml, respectively. The leaf extract showed the better DPPH free radicals scavenging activity than seed extract when compared with standard BHT that is shown in Figure 1(b).

### 3.3. Hemagglutination Activity

**Figure 2:**
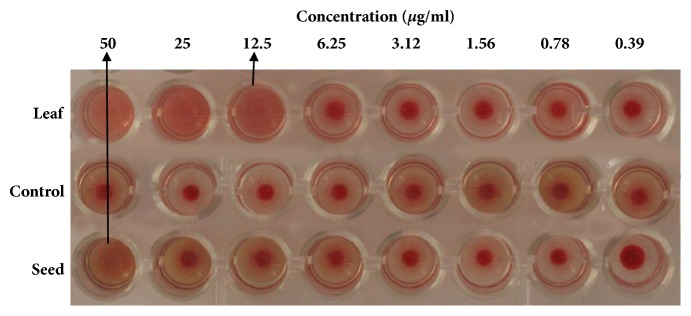
Hemagglutination activity of leaf and seed of* B. alba*. Leaf extracts showed better hemagglutination activity on goat blood when compare to control (without extract).

Hemagglutination assay was carried out to find out the presence of lectin protein which is considered to have potent anticancer and growth inhibitory activity [11, 43]. The hemagglutination activity of leaf and seed extracts of* B. alba *was represented in Figure 2. Both the leaf and seed extract showed concentration dependent hemagglutination activity on goat blood. In the present study, the leaf extract is able to agglutinate 2% blood at minimum 12.5*μ*g/ml concentration whereas the minimum concentration of seed extract was 50.0*μ*g/ml to agglutinate goat blood. Therefore, the seed extract is required comparatively in higher concentration than leaf extract to agglutinate goat blood which indicates that leaf extract contains more lectin protein than seed extract.

### 3.4. Cytotoxicity Test

**Figure 3:**
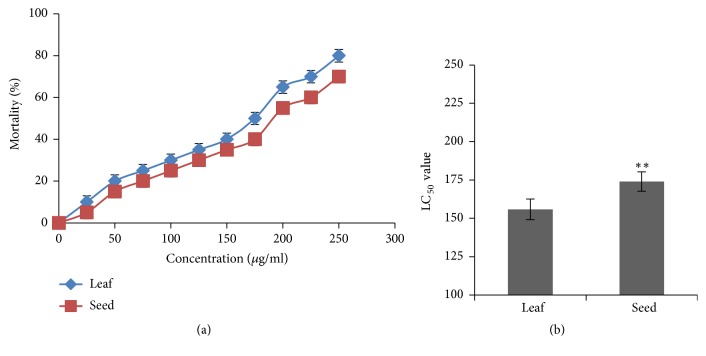
Cytotoxic activity of leaf and seed extract of* B. alba*. (a) Evaluation of LC_50_ values of leaf and seed extracts through regression line. (b) Comparison of LC_50_ values of leaf and seed extracts with each other. All the values are expressed as mean ± SD (n=3) and significance test was set up at P<0.05 (*∗*), P <0.01 (*∗∗*), and P <0.001 (∗∗∗).

In cytotoxicity assay, both leaf and seed extract of* B. alba *showed excellent activity which is the indication that they are biologically potent [44]. The 50% lethal concentration (LC_50_) values of each test sample were calculated from the corresponding regression equation and the LC_50_ values of leaf and seed extracts were 155.84±6.69 *µ*g/ml and 173.95±6.31*µ*g/ml, respectively, that is shown in Figure 3(a). The results evident that leaf and seed extract of* B. alba *exhibit notable toxic effects against brine shrimp nauplii. Leaf extract showed the better cytotoxic activity than seed extract (shown in Figure 3(b)) when compared with each other.

### 3.5. Evaluation of Weight Loss

**Figure 4:**
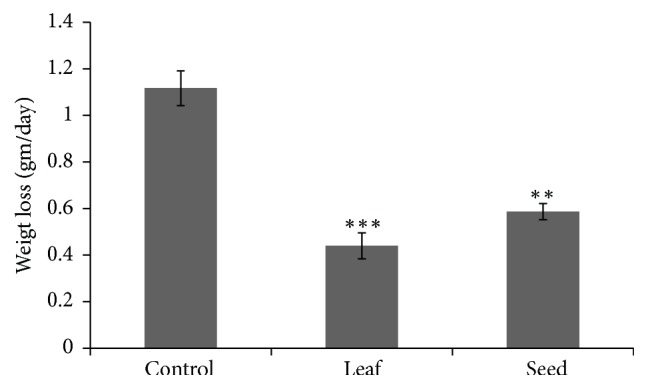
Evaluation of weight loss in response to leaf and seed extract in Swiss Albino mice. Data expressed as mean ± SD (n = 6) for all tested dosages. Significant differences of values are compared to values of control with leaf and seed samples and marked as *∗*p<0.05, *∗∗*p<0.01, and∗∗∗p<0.001.

The unexpected weight loss of body is a common symptom of cancer. The experimental mice lose their weight gradually after inoculation of cancer cells. The rate of weight loss values of control, leaf, and seed treated mice was evaluated as 1.12±0.07 gm/day, 0.45±0.05 gm/day, and 0.55±0.03 gm/day, respectively. The rate of weight loss of leaf treated mice was less than that of seed treated mice where control mice showed that the highest rate of weight loss which is represented in Figure 4.

### 3.6. Cell Growth Inhibition

**Figure 5:**
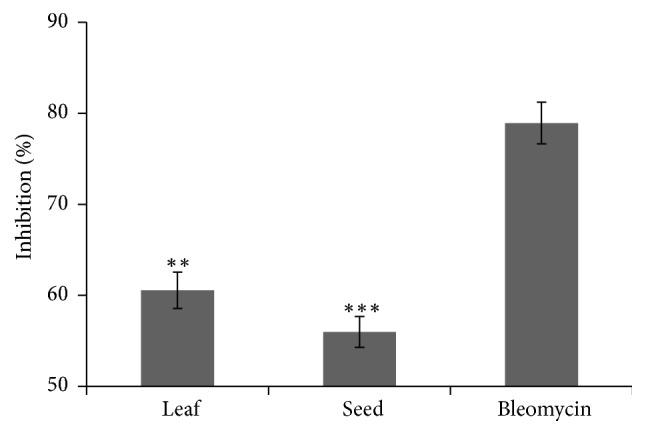
Comparison of cell growth inhibition of leaf and seed extract of* B. alba *along with standard (Bleomycin). A significant cell growth inhibition was observed in EAC cells in response to leaf and seed extract when compared with standard (Bleomycin). Data is expressed as mean ± SD (n = 3) for all tested dosages. Significant differences of values are compared to values of standard and various samples and marked as (*∗*p<0.05, *∗∗*p<0.01, and∗∗∗p<0.001).

Cell growth inhibition is widely used assay to determine the number of cells in a collective sample by using a hemocytometer which can easily separate the live cells from dead cell when using trypan blue dye exclusion [45]. The cells growth inhibitions after the administration of 5.00mg/kg/day leaf and seed extracts of* B. alba* are shown in Figure 5. The percentages of cell growth inhibition of leaf and seed extracts were calculated as 62.54±2.41% and 53.96±2.34 %, respectively, whereas inhibition by standard (Bleomycin) was 79.43±1.92 %. Hemocytometer counting of EAC cells using trypan blue showed that viability of EAC cells decreases considerably in all treated groups in comparison to control. According to the data presented in Figure 5, leaf extract shows relatively higher growth inhibitory activity than seed extract.

### 3.7. Study on Apoptosis

**Figure 6:**
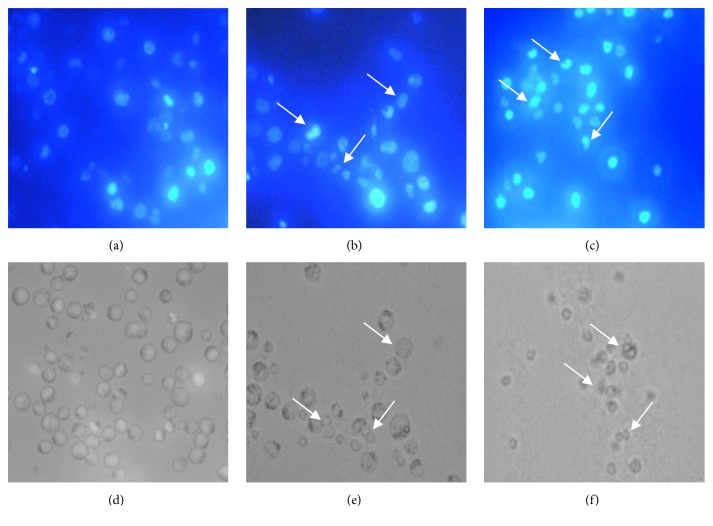
Fluorescence and optical microscopic observation of EAC cells for control mice and treated mice. (a), (b), and (c) represent as fluorescence microscopic view of control, leaf treated, and seed treated mice cell where (d), (e), and (f) express the optical microscopic view of control, leaf treated, and seed treated cells, respectively. Under both fluorescence microscope and optical microscope, normal and round shape nucleuses as well as cells appeared where cells from leaf and seed treated mice showed the nucleuses and cells having apoptotic properties that are indicated by white arrows.

Morphological changes of EAC cells collected from both control and treated mice were examined by Hoechst-33342 solution staining after scarification. The morphological alteration from both control and treated cells under fluorescence microscope and optical microscope were marked by arrow which is shown in Figure 6. Under fluorescence microscope, round, regular, and normal shaped nucleus were observed in cells from control mice whereas irregular, fragmented, and condensed nucleus were found in treated cells. Besides, under optical microscope, regular and normal shape cells were seen in control mice and shrinkage and segmented cells appeared in treated mice which were marked by arrow.

### 3.8. DNA Laddering

**Figure 7:**
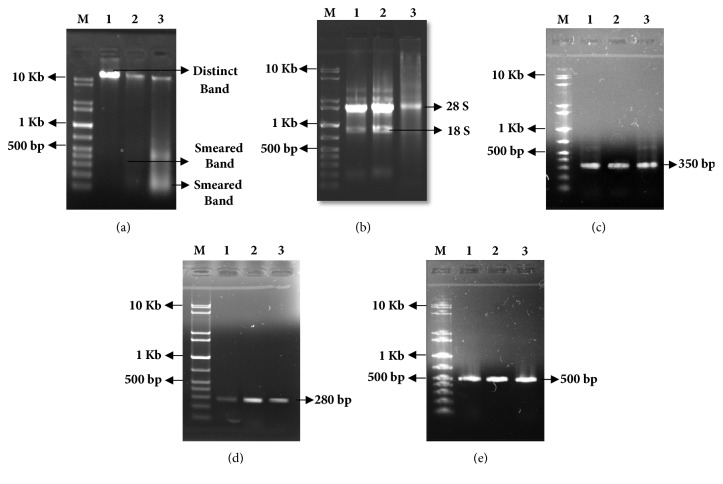
Agarose gel electrophoresis of extracted DNA, RNA, and PCR product from EAC cells treated with* B. alba* leaf and seed extracted and from control. Lane (M), (1), (2), and (3) represent as molecular marker, control, leaf treated, and seed treated, respectively. (a) Agarose gel electrophoresis of EAC cell's DNA for both control and treated mice which showed distinct band for control and smear for treated mice' mRNA. (b) The total RNA exhibited two different bands for both control and treated mice. (c) Expression of GAPDH in case of both control and treated EAC cells. (d) Upregulation of P^53^genes in treated cells in compare to control. (e) Downregulation of Bcl-2 gene in case of treatment compared to control.

After 6 days of treatment with* B. alba* leaf and seed extract, cells were collected from both control and treated mice and DNA was isolated that was run into 1% agarose gel electrophoresis. In the current investigation, distinct band was found on agarose gel for control mice's DNA whereas smeared and fragmented DNA bands were found in case of leaf and seed treated cell's DNA that are shown in Figure 7(a).

### 3.9. Gene Expression Analysis

For gene expression analysis, RNA was isolated from EAC cells aspirated from control and treated mice peritoneum and run into 1% agarose gel to observe the RNA quality. In the gel, both control and treated cell's RNA generated similar 28S and 18S bands at the same level that was shown in Figure 7(b). Quality and quantity of RNA were checked by nanodrop. The PCR amplification was set up using GAPDH primer which is a housekeeping gene. GAPDH was used as control and was amplified to check the successful conversion of mRNA into cDNA. In the current experiment, both the control and treated cells showed the identical bands at similar level at 350 bp position in the gel that is shown in Figure 7(c). Due to blessing of modern science and technology, many genes are discovered that are related to cancer as well as tumor development. The P^53^ and Bcl-2 are such kinds of genes which were taken for this investigation. P^53^ is one kind of tumor suppressor gene and its overexpression in treatment indicates inhibiting the growth of cancer cells [46]. In Figure 7(d), the expression of P53 gene was higher compared to control at the 280 bp position on the gel. On the other hand, the Bcl-2 is an antiapoptotic gene and its altered expression plays a critical role in regulating the cell's apoptosis [47]. In current investigation, the expression of Bcl-2 gene was lower compared to control at the 500 bp position on the gel which is shown in Figure 7(e). The results of gene amplification study reported that the treated mice showed upregulation of P^53^ mRNA and down regulation of Bcl-2 mRNA (when compared with their respective control) which indicated that the experimental extracts work to cause mitochondrial mediated apoptosis of EAC cells [13].

**Figure 8:**
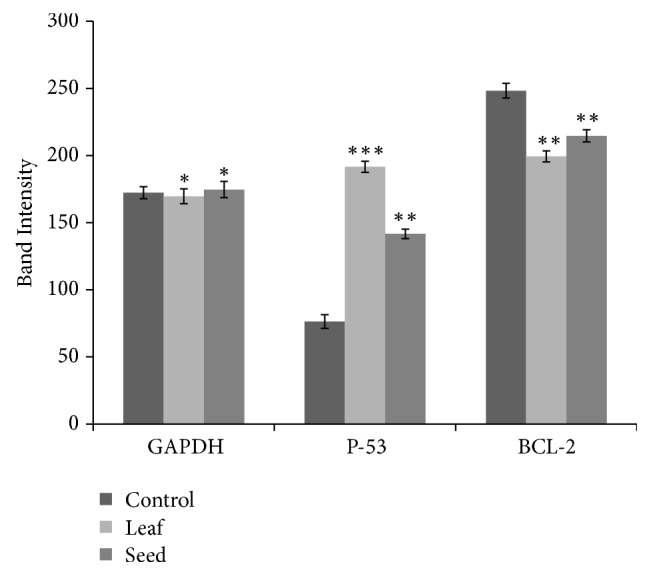
Gene expression level analysis of treated mice's mRNA with control based on their band intensity. About equal level of expression was found in GAPDH mRNA and upregulation of p53 and downregulation of antiapoptotic Bcl-2 were found in the treated cells compared to control. Data expressed as mean ± SD (n = 3) for all tested dosages. Significant differences of values are compared to values of control and treated samples and marked as (*∗*p<0.05, *∗∗*p<0.01, and∗∗∗p<0.001).

The relative band intensity of the genes was studied using software named Gelanalyzer-2010. The expression levels of GAPDH, P^53^, and Bcl-2 mRNA are shown in Figure 8 based on their band intensity. In GAPDH mRNA, near to equal level of expression was seen in both control and treated EAC cells. The treated cells showed higher expression level than control in P^53^ mRNA but, in Bcl-2 mRNA, treated mice showed less expression level than control. The band intensity of GAPDH, P-53, and Bcl-2 mRNA for control, leaf, and seed treated mice was evaluated as 172.33±4.51, 169.67±5.13, 174.67±5.50; 76.33±5.51, 191.67±4.16, 141.67±4.04; and 248.33±6.03, 199.33±3.51, 214.67±4.51, respectively.

## 4. Discussion

Cancer is one of the most fatal diseases over the world with high rate of mortality but the success in its treatment option is not much satisfactory [13]. Therefore, scientist as well as pharmaceutical companies are in constant search of safer natural compound to treat cancer. Our current experimental plant* B. alba* showed various activity as anticancer agent which was proved by different bioassay like antioxidant activity, hemagglutination activity, cytotoxic activity, and cell growth inhibition assay. Our molecular studies also suggest that the leaf and seed extracts of* B. alba* can inhibit EAC cell growth.

It was more or less established that having antioxidant activity of an extract or a specific compounds exhibits anticancer activity in a definite mechanism [48]. Our results suggest that the both leaf and seed extracts of* B. alba* have significant antioxidant activity but the leaf extract showed the better activity than the seed extract when compared with standard (BHT). Lectin protein also has a great role in anticancer activity which causing the apoptosis and cell growth inhibition and it was proved by several* in vitro* and* in vivo *studies [11]. Hemagglutination activity assay was conducted for detection of lectin protein in the experimental plant. In this assay the leaf and seed extracts agglutinated the goat blood (shown in Figure 2) which confirm the presence of lectin protein.

The brine shrimp lethality bioassay was used to predict the cytotoxic activity. It has been reported that any compound having a LC_50_value less than 250*µ*g/ml is considered significantly active and is suitable candidate for further research [49]. A considerable cytotoxic activity was found in the investigated leaf and seed extracts which was measured as (LC_50_ value) 155.84±6.69*µ*g/ml and 173.95±6.31*µ*g/ml, respectively, that is shown in Figure 3. The experimental extracts obstructed the unexpected weight loss of cancerous mice which gave also an important signal about the plant's activity. The rate of weight loss of leaf treated and seed treated mice was less than the control mice and that was determined as 1.12±0.07 gm/day, 0.45±0.05 gm/day, and 0.55±0.03 gm/day, respectively, which is shown in Figure 4.

Due to the presence of substantial antioxidant activity, hemagglutination activity, and cytotoxicity, the plant extracts were able to inhibit the growth of EAC cell significantly. The EAC cells are experimental tumor models used worldwide in cancer research [50]. The leaf extract exhibited the higher cells growth inhibition than seed extract where an anticancer drug Bleomycin was used as standard. The leaf and seed extracts not only inhibited the cells growth but also represent morphological features of apoptosis. In Figure 6, fluorescence and optical microscopy of EAC cells from the mice treated with leaf and seed extract showed significant morphological changes including cell membrane blebbing, cells shrinkage, chromosomal condensation, and nuclear fragmentation occurred in nucleus, whereas normal and round size and shape cells and regular nucleus were seen in control mice. These morphological alterations indicate the apoptosis of EAC cells which is the common way to expel the inappropriate and redundant cells from body through a series of processes without damaging any normal cells. Lacking of apoptosis process plays a vital role in tumor development that lead to abnormal cell proliferation as well as cancer development [51]. Cells and nuclear shrinkage, chromatin condensation, and formation of apoptotic bodies were found in this experiment which is thought to be an efficient indicator for any kinds of cancer treatment and prohibition. The cell apoptosis was confirmed by performing by a reliable way called DNA laddering which is a biochemical hallmark of apoptosis [52]. Smeared and fragmented bands were shown in 1% agarose gel electrophoresis for leaf and seed treated mice's DNA but the DNA extracted from control mice showed a unique and distinct band. This result indicates that the experimental plant extracts have a notable ability to degrade the DNA which is shown in Figure 7.

Molecular studies demonstrate that apoptosis is an ideal process to detect cell death into the body which is controlled by some regulatory proteins like Bcl-2, Bcl-X, BAX, BAK, P^53^, Caspase-3,8,9, etc. The downregulation of Bcl-2 and upregulation of P^53^ mRNA represent mitochondria mediated cell apoptosis [13]. A previous study reported that the various phytochemicals like flavonoids, alkaloids, glycosides, etc. are responsible for inducing upregulation of P^53^ gene that can cause the DNA damage in cancer cells [53]. In this investigation, the expression pattern and level of P^53^ and Bcl-2 mRNA of leaf and seed treated mice were evaluated along with control. The upregulation of P^53^ mRNA and downregulation of Bcl-2 mRNA (shown in Figures 7(d), 7(e) and 8) were a reliable indication of mitochondrial apoptosis of EAC cells. The results of cell growth inhibition, observation of cellular morphology, and DNA laddering also suggest a strong support in alteration of genetic construction as well as apoptotic death of EAC cells which were treated by leaf and seed extracts of* B. alba*. Therefore, we strongly believe that the various phytocompounds present in the extract of* B. alba* have potential anticancer activity. Further studies on detection of bioactive compounds that are responsible for inhibition of EAC cell growth will play a great therapeutic role in modern medicine.

## 5. Conclusion

Cancer is a devastating fatal disease all over the world and scientists are still trying to find out an effective way to combat this disease. This study reported that* B. alba* plant is a natural source of different bioactive phytonutrients having significant antioxidant, cytotoxic, hemagglutination, and growth inhibition activity against cancer cells. The current investigation provides a solid evidence that the leaf and seed extracts of* B. alba* contain a significant antiproliferative features with well-known massive health benefit. The findings of the current project can further be investigated for discovering anticancer drug lead compounds.

## Figures and Tables

**Table 1:** The sequence of primers used for PCR amplification.

Gene Name	Primer Sequence	Gene Accession number
GAPDH	Forward: 5′-GTGGAAGGACTCATGACCACAG - 3′ Reverse: 5′-CTGGTGCTCAGTGTAGCCCAG - 3′	NM_002046

P^53^	Forward: 5′-CACAAAAACAGGTTAAACCCAG - 3′ Reverse: 5′-AGCACATAGGAGGCAGAGAC - 3′	Ref: Sara *et al.* [42]

Bcl-2	Forward: 5′-GTGGAGGAGCTCTTCAGGGA- 3′ Reverse: 5′-AGGCACCCAGGGTGATGCAA- 3′	NM_000633

**Table 2:** Phytochemical screening of leaf and seed extracts of *B. alba*.

Sl. No.	Name of Compounds	Leaf	Seed
01	Carbohydrates	+	+

02	Proteins	+	+

03	Fats	+	+

04	Glycosides	+	+

05	Alkaloids	+	+

06	Tannins	+	+

07	Phlobatannins	-	-

08	Flavonoids	+	+

09	Steroids	+	+

10	Saponins	+	+

11	Phenolic Compounds	+	+

12	Phytosterols	+	+

## Data Availability

All the data presented in the current paper will be readily available from me upon request (reza.gen@ru.ac.bd).
